# City Hospital of Philadelphia

**Published:** 1859-09

**Authors:** 


CITY HOSPITAL OF PHILADELPHIA.—Dr. John Bell has been appointed, by the new Board of Health, Physician to this institution.

